# Ion channels in cancer-induced bone pain: from molecular mechanisms to clinical applications

**DOI:** 10.3389/fnmol.2023.1239599

**Published:** 2023-08-17

**Authors:** Huan-Jun Lu, Xiao-Bo Wu, Qian-Qi Wei

**Affiliations:** ^1^Institute of Pain Medicine and Special Environmental Medicine, Nantong University, Nantong, China; ^2^Department of Infectious Diseases, General Hospital of Tibet Military Command, Xizang, China

**Keywords:** chronic pain, cancer-induced bone pain, ion channels, antinociception, clinical application

## Abstract

Cancer-induced bone pain (CIBP) caused by bone metastasis is one of the most prevalent diseases, and current treatments rely primarily on opioids, which have significant side effects. However, recent developments in pharmaceutical science have identified several new mechanisms for CIBP, including the targeted modification of certain ion channels and receptors. Ion channels are transmembrane proteins, which are situated on biological cell membranes, which facilitate passive transport of inorganic ions across membranes. They are involved in various physiological processes, including transmission of pain signals in the nervous system. In recent years, there has been an increasing interest in the role of ion channels in chronic pain, including CIBP. Therefore, in this review, we summarize the current literature on ion channels, related receptors, and drugs and explore the mechanism of CIBP. Targeting ion channels and regulating their activity might be key to treating pain associated with bone cancer and offer new treatment avenues.

## 1. Introduction

According to the International Association for Pain Research, pain is defined as an unpleasant feeling or emotion that arises from actual or potential tissue damage (Raja et al., 2020). The process of pain development involves signal transduction from peripheral pain receptors, further signal transduction in the spinal cord, brain stem, and thalamus, and the processing of pain signals in the cerebral cortex (Yam et al., 2018). Pain can be classified as acute or chronic according to the duration of pain symptoms. Acute pain tends to be relieved by the removal of the injurious stimulus, whereas chronic pain persists constantly or intermittently (Cohen et al., 2021). Owing to its complicated mechanism of generation, chronic pain is more difficult to treat than acute pain. Cancer pain is a prevalent symptom among patients with cancer, with approximately 30–50% of patients experiencing chronic pain during clinical treatment (Broemer et al., 2021). Moreover, chronic pain can persist in cancer survivors after treatment, along with other symptoms, such as anxiety, depression, fatigue, and loss of appetite, severely affecting their daily function (Poço Gonçalves et al., 2021). Cancer-induced bone pain (CIBP) is one of the most common types of chronic pain experienced by patients with advanced cancer (Kapoor et al., 2021). Although the incidence of primary bone cancer is low, most tumors can metastasize to the bone tissue, causing severe pain in the bone tissue. The mechanism of bone cancer pain is complex and involves two main aspects: the influence of chemical media, such as changes in the local microenvironment of bone tissue; and mechanical deformation, such as the mass effect of the tumor and the resulting pressure, which results in the activation of stimulation receptors in the endosteum (Zheng et al., 2022). The current three-stage therapy for CIBP, advocated by the World Health Organization, involves the use of non-body anti-inflammatory and analgesic drugs or opioids based on pain severity (Wiffen et al., 2017; Chapman et al., 2020). However, opioid drugs often cause addiction and other side effects such as itching, gastrointestinal problems, sleep disturbances, and cognitive issues (Kata et al., 2018; Volkow and Blanco, 2020). Therefore, elucidating the mechanism of bone cancer pain and determining more effective analgesic methods are crucial.

Ion channels, which are specialized proteins located on the membranes or organelles, form highly selective pores on the phospholipid bilayer, allowing charged ions of appropriate size to pass through *via* passive transport. Ion channels are widely distributed in various tissues and organs and participate in various physiological and pathological processes (Subramanyam and Colecraft, 2015; De Logu and Geppetti, 2019). Previous phytochemical and pharmacological investigations demonstrated that ion channels play an important role in pain regulation (Raouf et al., 2010; Moore et al., 2018; Bouali-Benazzouz et al., 2021). Somatic neurons involved in pain transmission are present in the dorsal root ganglion (DRG). In these neurons, various ion channels play important roles in the transmission of pain sensations. For example, transient receptor potential vanilloid (TRPV1) in the DRG is mainly expressed in nociceptors and sensory neurons and is involved in the regulation of heat and inflammatory pain (Karai et al., 2004; Chen et al., 2022; Katz et al., 2023). Increased voltage-gated sodium channel 1.7 (NaV1.7) expression leads to increased neuronal excitability and ultimately causes neuropathic pain in animal models (Li et al., 2018). In the early stage of nerve injury, the expression of the Cav3.2 channel and functional enhancement of damaged neurons lead to an increase in ectopic discharge frequency (Fayad et al., 2022). Transient receptor potential ankyrin 1 (TRPA1) and transient receptor potential melastatin 8 (TRPM8) knockout mice demonstrated relief of pain sensitization in neuropathic models (Trevisan et al., 2016; de Caro et al., 2018). The mechanical sensitization of pain in neuroinflammation has been reported to be mediated by the pressure-sensitive piezo ion channel family (Murthy et al., 2018). Ion channels are often the sites of action of drugs or toxins (Bagal et al., 2013). Therefore, as targets of analgesic drugs, ion channels have always been the focus of research and direction of new drug development. This review provides an overview of the functional mechanisms of various ion channels in CIBP. In addition, we discuss their potential application in formulating therapeutic strategies to address challenges in cancer pain treatment in the future.

## 2. Ion channels participating in CIBP–TRP channel family

The TRP family comprises a group of nonselective cationic ion channels found in mammals. This family includes 28 members categorized into six subfamilies: TRPV (vanilloid), TRPC (carnical), TRPP (polycystin), TRPA (ankyrin), TRPM (melastatin), and TRPML (mucolipin). The TRP channel has a pore structure between S5 and S6, forming a cationic channel that is mainly permeated by calcium, sodium, and magnesium ions (Huang et al., 2020). TRP channels are widely distributed in both the peripheral and central nervous systems and act as molecular receptors that can be activated by various physical and chemical stimuli, such as pH changes; chemical irritants, such as capsaicin and mustard; and changes in the temperature and osmotic pressure (Ramsey et al., 2006). Some TRP channels also act as thermoreceptors in peripheral sensory neurons. For example, TRPV1, TRPV2, and TRPA1 are activated by high temperatures (>39°C), while TRPM8 functions as a cold sensor activated by cool temperatures (<15°C). These channels are activated in specific temperature ranges, which makes them important for temperature sensing (Cabañero et al., 2022). Additionally, some TRP family members are activated by various intracellular signaling molecules, such as inflammatory mediators, arachidonic acid and its metabolites, lipoxygenase products, and adenosine (Nishida et al., 2015).

TRP channels mediate diverse physiological functions as they can be activated by different external stimuli. For instance, TPRV1 is a temperature receptor that can be activated by heat and capsaicin, whereas some members of TRP, such as TRPN channels, are mechanically sensitive ion channels that can transduce mechanical signals from the extracellular space to the intracellular space by sensing changes in stress on the cell membrane surface (Montell, 2001), which allows them to regulate hearing and touch in fruit flies and mammals. Mechanical conduction occurs mainly in the vascular endothelium, muscles, joints, skin, and other sensory cells. Transient Receptor Potential Vanilloid 4 (TRPV4) is expressed in cochlear hair and bone cells, where it regulates mechanical stress (Liedtke, 2005). The TRP family of proteins plays a crucial role in pain transduction. For example, TRPA1 is involved in the conduction of mechanical sensation in afferent-innervated blood vessels and mucosal tissues of the colon, with high and low threshold values (Jain et al., 2020). TRPV4 and TRPA1 knockout can inhibit the generation of painful behaviors in pancreatitis (Ceppa et al., 2010). Transient receptor potential vanilloid 3 (TRPV3), which is mainly expressed in keratinocytes of the skin and is structurally similar to TRPV1, is involved in temperature perception and thermal pain (Moqrich et al., 2005). TRPV3 activation can also regulate skin barrier formation, germatogenesis, wound healing, and prostaglandin E2 (PGE2) release, resulting in heat pain and pain hypersensitivity when overexpressed in keratinocytes (Um et al., 2022).

### 2.1. TRPV1

TRPV1, also known as the capsaicin receptor, is a peripheral temperature receptor that is widely distributed in nociceptive receptors and is involved in acute and chronic inflammatory pain. It is specifically expressed in the small-and medium-sized neurons of the DRG and the first and second layers of the spinal dorsal horn (Ma et al., 2022). In a rat model of Complete Freund’s adjuvant (CFA)-induced inflammatory pain, the TRPV1 expression levels in the dorsal horn of the spinal cord were significantly increased. However, the intraspinal injection of a TRPV1-specific antagonist can block heat and mechanical pain sensitivity in a CFA model (Yang et al., 2014). Furthermore, TRPV1 expression also appears in large-and medium-diameter neurons of the DRG in chronic pain states, which are believed to mediate mechanical touch and explain mechanical sensitivity in chronic inflammatory pain (Yu et al., 2008). Recent research has demonstrated that CIBP includes not only the progression of inflammatory pain but also neuropathic pain, which has unique characteristics (Zheng et al., 2022). Qian et al. (2021) reported that the expression of ASIC3 and TRPV1 was significantly increased in the L4-L6 DRG of CBIP model mice. Ghilardi et al. found that the selective blockade of TRPV1 attenuates bone cancer pain by detecting TRPV1 on sensory neuron fibers that innervate the mouse femur. Furthermore, the administration of a TPRV1 antagonist or knockdown of the TRPV1 gene resulted in significant attenuation of nociceptive behaviors (Ghilardi et al., 2005). In a more recent study, the employment of RNA interference technique to knock down TRPV1 resulted in increased mechanical threshold and paw withdrawal latency in rats. Furthermore, the impact of pain-inducing agent, such as class I histone deacetylases and TNFα, was attenuated in the spinal cord of these TRPV1 knockdown rats (Zhang et al., 2019). Similarly, in a TRPV−/− mice model, bone pain and sensory neuron excitation are significantly decreased (Wakabayashi et al., 2018). These results suggest the importance role of TRPV1 in algesia and hypersensitivity.

### 2.2. TRPA1

TRPA1, also known as ANKTM1, is an ion channel that transports calcium ions into cells after activation. As a TRP family member, TRPA1 has certain similarities with TRPV1, including a structure consisting of six transmembrane domains with intracellular N-and C-termini. TRPA1 is widely expressed in various organs including the brain, DRG, heart, pancreas, and gastrointestinal tract (Nilius et al., 2012). In the DRG, TRPA1 is mostly expressed in small-sized neurons and is considered a noxious cold sensor as it can be activated by harmful temperatures lower than 17°C (del Camino et al., 2010). Unlike TRPV1, TRPA1 inhibition by the administration of A1 antagonists did not change the body temperature in preclinical studies (Koivisto et al., 2022). Thus, TRPA1 is currently considered a target for cold hypersensitivity in the animal models of chronic pain. In addition to cold temperatures that activate TRPA1, the receptor is also activated by a variety of natural compounds such as allicin (from garlic), allyl isothiocyanate (from wasabi and mustard), and carvacrol (from oregano and thyme). TRPA1 is gated by metabolites [reactive nitrogen species (NO), cyclopentenone prostaglandins, and methylglyoxal] during oxidative stress progression and tissue damage (Nilius et al., 2012). Because some TRPA1 agonists are related to the inflammatory and chronic pain processes, pharmacological regulation of TRPA1 is considered an important analgesic therapy. Recent research has suggested that TRPA1 may be involved in the development and maintenance of CIBP (Liu et al., 2021). In animal models of CIBP, TRPA1 expression was upregulated in the DRG and spinal cord, and TRPA1 activation contributed to pain hypersensitivity (Liu et al., 2021). Another study reported that intrathecal administration of a TRPA1 antagonist attenuated mechanical allodynia and thermal hyperalgesia in rats with bone cancer-induced pain (Jin et al., 2023). In addition to its role in pain signaling, TRPA1 has been implicated in the regulation of bone metabolism. *In vitro* studies have demonstrated that TRPA1 activation can stimulate osteoclast differentiation and function, leading to bone resorption (Nummenmaa et al., 2020). Collectively, these findings suggest that the TRP channel family is a promising target for CIBP treatment. However, further research is warranted to completely understand the role of these channels in CIBP and develop more specific and effective TRP-targeted therapies.

### 2.3. TRPV4

TRPV4, which is a calcium-permeable ion channel expressed in the sensory neurons, plays an important role in pain perception (Hu et al., 2023). TRPV4 has been shown to contribute to the development and maintenance of pain in CIBP (Wang et al., 2015; Xu et al., 2023). TRPV4 is expressed in the sensory neurons that innervate the bone tissue, and its activation can lead to the release of neurotransmitters that signal pain. TRPV4 is upregulated in the sensory neurons that innervate the bone and contribute to pain development (Xu et al., 2023). Blocking TRPV4 with specific inhibitors or genetic manipulation can reduce pain in animal models of CIBP (Xu et al., 2023). In addition to its role in pain perception, TRPV4 has been implicated in the development of bone metastases. TRPV4 is expressed in osteoclasts, which break down bone tissue. TRPV4 activation in osteoclasts leads to increased bone resorption, which contributes to the development of bone metastases (Das and Goswami, 2022). Studies have shown that blocking TRPV4 in osteoclasts can reduce bone resorption and inhibit bone metastases (Masuyama et al., 2008). Overall, TRPV4 plays an important role in CIBP and development of bone metastases. Targeting TRPV4 may provide a new therapeutic approach for the management of CIBP and the prevention of bone metastases.

### 2.4. ASICs

ASICs belong to the osteosin/epithelial sodium channel superfamily and are highly expressed in the mammalian nervous system. They can detect changes in the pH of the internal environment and play a regulatory role in response to such changes (Cheng et al., 2018). Alterations in the pH of the internal environment can activate ASICs under both physiological and pathological conditions. For instance, in bone tumor metastasis, the primary cause of bone destruction is the effect of osteoclasts, which results in bone cancer pain. Monocytes accumulate on the surface of mineralized bone, and the surrounding acidic microenvironment is maintained (Ahmad et al., 2018). A decrease in pH can lead to the excitation of neurons, resulting in a larger amplitude of slow activation and inactivation of the inward current (Duzhyy et al., 2021).

ASIC3 is the most pH-sensitive ion channel in the ASICs family subtype, detecting a narrow range of acidic pH (6.7–7.3; Lee and Chen, 2018). Studies have shown that in rat models of CIBP, the expression of ASIC3 mRNA and protein is upregulated on the same side of the DRG, and when ASIC3 is upregulated, the pain threshold simultaneously decreases (Qian et al., 2021). Therefore, the upregulation of ASIC3 may be a potential factor in the development of pain in bone cancer. Increased osteoclast activity is a pathological marker of bone cancer, leading to bone remodeling and sensitization of nerve nociceptors and resulting in bone cancer pain (Andriessen et al., 2021). The inhibition of ASIC3, which responds to the bone stroma-degrading proton H1 secreted by osteoclasts, attenuates pain-related behavior in bone cancer pain models (Morgan et al., 2020). In addition to ASIC3, the other two subtypes of the ASIC family, ASIC1 and ASIC2 in the ASICs family are also involved in pain progression (De Logu and Geppetti, 2019). In the CIBP model, the ASIC1, ASIC2, and ASIC3 expression levels are upregulated. After treatment with opioids, while pain behavior was alleviated, the ASIC1, ASIC2, and ASIC3 expression decreased in the treatment group (Heo et al., 2017), suggesting that the ASICs family of channels may also be potential targets for cancer pain treatment.

### 2.5. Piezo channels in CIBP

Piezo channels are a family of ion channels that respond to mechanical stimuli and are involved in the conversion of mechanical signals into electrical signals. They are widely expressed in various tissues and have been implicated in various physiological and pathological processes including pain sensation (Kefauver et al., 2020). Recent research has suggested that piezo channels play a role in CIBP, a common and debilitating symptom of bone metastases (Qin et al., 2021). Bone metastases occur when cancer cells from primary tumors spread to the bones, leading to bone destruction and pain. Piezo1 and Piezo2 are expressed in sensory neurons and bone cells, including osteoblasts and osteoclasts, suggesting their involvement in this process. Additionally, Piezo1 was upregulated in an osteoblast model and mechanical stimulation of the bone caused an increase in Piezo1 expression, leading to the release of inflammatory cytokines that sensitize sensory neurons and contribute to pain sensation (Wang et al., 2020). These findings suggest that Piezo channels contribute to CIBP by transducing mechanical stimuli into electrical signals that activate sensory neurons and promote inflammation.

### 2.6. P2X receptors

P2X receptors are a family of ion channels activated by extracellular ATP. Seven members of the P2X receptor family (P2X1–P2X7) are mainly expressed in the neurons and glial cells (North, 2002). P2X1–6 receptors are mainly found in the dorsal root ganglia, ganglia nodal ganglia, and trigeminal ganglia, whereas P2X7 is mainly found in immune system cells. These receptors have been implicated in various physiological and pathological processes including pain sensation (North, 2002). In a bone cancer pain model, ATP-mediated purine signaling demonstrated to play a crucial role in the occurrence of cancer pain (Zhang et al., 2020). ATP acts as a ligand for the P2X receptor, promoting the production of inflammatory cytokines by the nerves and immune cells. In an MRMT-1-induced bone cancer pain model, ATP was released into the neurons and astrocytes to activate the P2X receptors that mediate pain-related behaviors (Falk et al., 2019). P2X3 and P2X7 are the two main members of the P2X receptor family that have been implicated in CIBP (Zhang et al., 2020). P2X3 is expressed in sensory neurons, whereas P2X7 is expressed in bone cells including osteoblasts and osteoclasts. In a mouse model of bone metastases, P2X3 was upregulated in sensory neurons, and blocking P2X3 with a specific antagonist reduced pain behavior (Hansen et al., 2012). Another study found that P2X7 was upregulated in osteoclasts in CIBP, and the activation of P2X7 in osteoclasts led to the release of inflammatory cytokines that sensitized sensory neurons and contributed to pain sensation (Hansen et al., 2011). These findings suggest that P2X receptors may play a role in CIBP by promoting inflammation and sensitizing sensory neurons. Targeting P2X receptors may be a potential strategy for the development of new therapies for CIBP. However, more research is warranted to completely understand their roles in this process and develop effective P2X-targeted therapies.

## 3. Peripheral and central mechanism in CIBP

**Figure 1:**
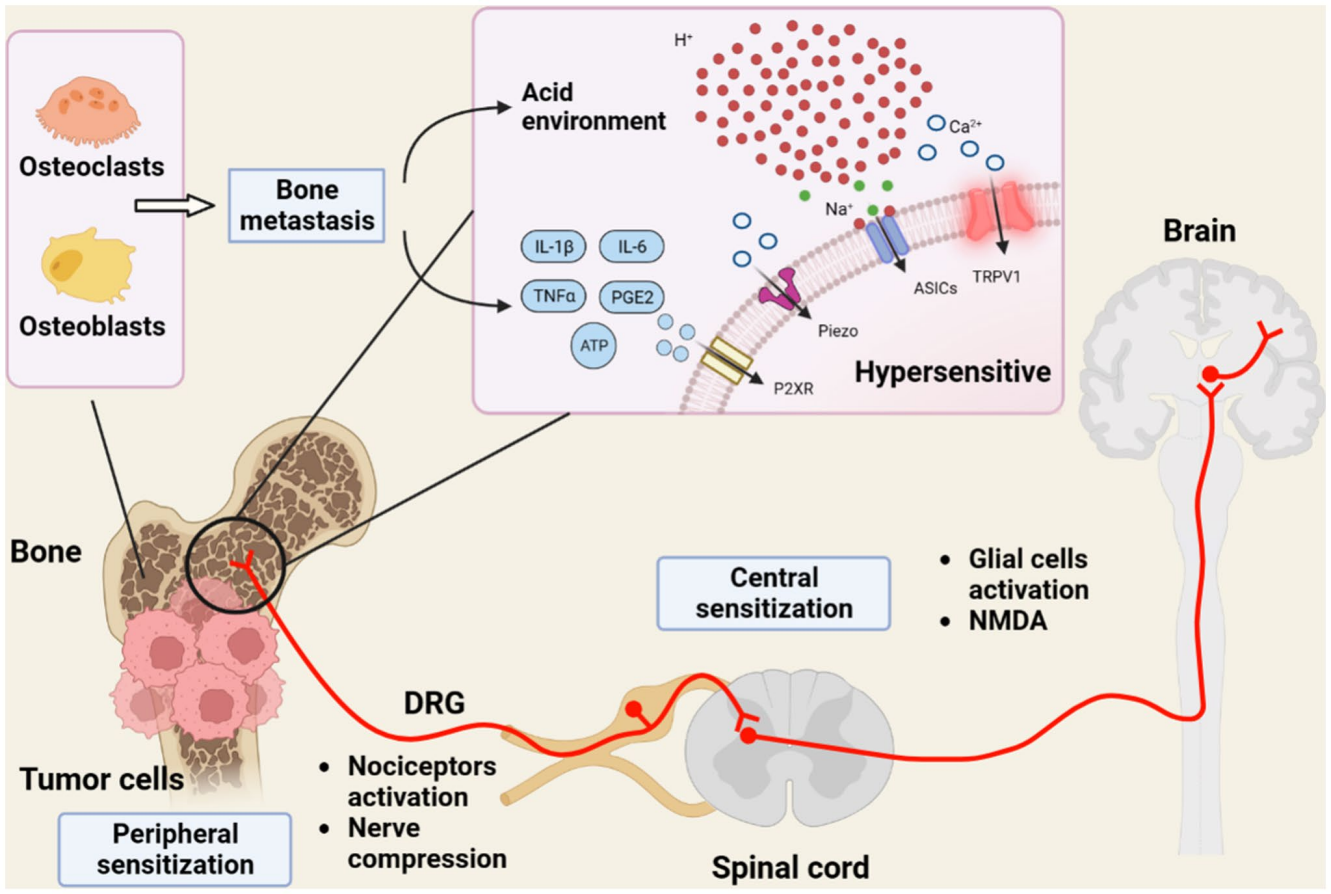
Diagram showing the mechanism of chronic pain progression in bone tumor. Tumor growth and bone destruction disrupt the balance between osteoblasts and osteoclasts, leading to bone metastasis. The acidic environment created by tumor cells and inflammatory mediators activates receptors and ion channels in both nociceptors and the dorsal root ganglion (DRG) neurons. In the central nervous system, the reactive glial cells and the expression of receptors on postsynaptic neurons participate in cancer-induced bone pain (CIBP). As a result, peripheral and central sensitization contribute to hypersensitivity and pain development. This figure was created with BioRender.com.

CIBP is a multifaceted phenomenon that involves various mechanisms in the peripheral and central nervous systems that regulate CIBP development and progression (Luger et al., 2005). Notably, the mechanisms underlying CIBP are complex and can vary based on the type and stage of cancer. Therefore, understanding these mechanisms is vital for the development of effective treatment strategies to help manage CIBP and enhance the patient’s quality of life. In the following test, we delved into some of the peripheral and central mechanisms that play significant roles in CIBP (Figure 1).

### 3.1. Peripheral mechanism

Sensory neurons establish neural connections with various bone structures, including the bone marrow, mineralized bone, and periosteum (Tabarowski et al., 1996; Mach et al., 2002). Traditionally, the involvement of sensory neurons in CIBP development has been associated with mechanical strain experienced by bone tissues. Noxious stimuli, primarily detected by Aδ fibers and C-fibers, are transmitted from sensory neurons to the DRGs and subsequently relayed to the brain. The presence of tumor cells within the bone marrow leads to their proliferation and consequent disruption of sensory fiber distribution, accompanied by electrophysiological alterations in the sensory neurons (Peters et al., 2005; Haroun et al., 2022; Yoneda et al., 2023). Notably, a diverse array of nociceptors, ion channels, and receptors is distributed among sensory neurons, osteoclasts, osteoblasts, and immune cells. These molecular entities facilitate the detection of protons, reception of cytokine signals, and transduction of mechanical stress, thereby facilitating reciprocal communication with tumor cells (Hofbauer et al., 2021). Consequently, this bidirectional interplay culminates in the formation of a bone tumor microenvironment.

Inflammatory mediators play a pivotal role in the peripheral mechanisms of CIBP (Habberstad et al., 2022). These mediators are released by cancer and bone cells with tumor growth and bone destruction and can contribute to pain sensation through various mechanisms. Inflammatory mediators include cytokines, chemokines, and prostaglandins. These molecules can activate receptors in the sensory neurons, leading to the release of neuropeptides that promote inflammation and pain (Lu and Gao, 2023). Tumor necrosis factor (TNF)-α is a cytokine that is upregulated in bone metastases and contributes to CIBP by activating TRPA1 in the sensory neurons, leading to the release of substance P and other neuropeptides that promote inflammation and pain (Zhao et al., 2019). Other inflammatory mediators implicated in CIBP include Interleukin-1β (IL-1β), IL-6, and PGE2. IL-6, for instance, induces the functional upregulation of TRPV1 in DRG neurons through the activation of the JAK/PI3K signaling pathway, contributing to the pathogenesis of bone cancer pain (Fang et al., 2015). These molecules can also activate receptors in the sensory neurons and promote inflammation, pain, and sensitization of nociceptors, leading to increased pain sensitivity (Adamopoulos, 2018). In contrast, inflammatory mediators contribute to bone destruction and nerve compression by disrupting the balance between bone resorption and formation (Zhen et al., 2022). IL-1β and TNF-α can promote osteoclast activation and bone resorption, leading to the release of calcium ions and other bone matrix components that can activate nociceptors and contribute to pain sensation (Nguyen et al., 1991). Numerous members of the TRP channel family, such as TRPV1, TRPV4, and TRPV6 exhibit calcium permeability, rendering them sensitive to fluctuations in the extracellular calcium levels. Consequently, alterations in the calcium concentration within the extracellular space can activate these receptors, thereby instigating CIBP (Den Dekker et al., 2003; Hagenacker et al., 2008; Lee et al., 2016). Notably, the bone microenvironment contains various factors that contribute to nociceptor activation and sensitization. Neurotrophins (e.g., nerve growth factor and Brain Derived Neurotrophic Factor) and ATP, which originate from cancer cells or nerve damage, have been implicated in this process (Aielli et al., 2019; Haroun et al., 2022). ATP acts as a dual stimulus for P2X receptors, eliciting responses from both sensory neurons and osteoclasts, making it a crucial mediator of algesia.

Under physiological conditions, the bone marrow exhibits a sinusoidal structure perfused by a combination of arterial and venous blood, thereby establishing an inherently hypoxic environment within the bone marrow (Harrison et al., 2002). Tumor cells, characterized by a distinct metabolic profile primarily centered on aerobic glycolysis, commonly referred to as the “Warburg effect, “contribute to the accumulation of protons and lactate production in the hypoxic milieu (Maes et al., 2012; Ganapathy-Kanniappan and Geschwind, 2013). Consequently, acidification of the microenvironment affects the behavior of both osteoblasts and osteoclasts, stimulating resorptive activity while impeding mineralization (Arnett, 2010). Osteoclasts facilitate the release of protons into the resorption lacuna *via* a vacuolar ATPase (V-ATPase) mechanism. Subsequently, these protons have the potential to escape into the bone marrow microenvironment, either owing to inadequate sealing of the lacuna or as a consequence of osteoclast apoptosis (Cappariello et al., 2014).

As discussed previously, TRP channels and ASICs exhibit heightened sensitivity to fluctuations in the pH levels. Within the context of bone marrow innervation, nociceptors display an abundant expression of TRP channels and ASICs, with a notable prevalence of TRPV1(Haroun et al., 2022). Interestingly, sensory neurons innervating tumor-bearing bones maintain elevated TRPV1 expression levels even in the presence of tumor-induced injuries (Ghilardi et al., 2005). The effect of tumors on TRPV1 expression in neurons may be multifaceted. Tumor inoculation in the mouse femur led to a discernible increase in the ipsilateral TRPV1 expression within the DRGs (Niiyama et al., 2007). In a human multiple myeloma model, Hiasa et al. demonstrated that JJN3 cells in collaboration with osteoclastogenic cytokines foster an acidic bone microenvironment that subsequently triggered bone pain through the excitation of ASIC3-activated sensory neurons. This effect was effectively inhibited by the application of a selective ASIC3 antagonist (Hiasa et al., 2017). Despite the chemical changes shaping the acidic bone microenvironment, physical tumor expanding growth also induces CIBP. As tumors grow in the bone, they cause mechanical stress to the bone tissue, leading to microfractures and bone pain. Piezo channels can be activated by mechanical stress, leading to pain sensation (Qin et al., 2021).

In contrast, tumor growth increases the distribution of sensory neurons. This process, known as perineural invasion (PNI), occurs when cancer cells invade the epineurial, perineurial, and endoneurial spaces of the neuronal sheath, resulting in dense nerve innervation in the tumor tissue (Yoneda et al., 2021). Recent studies have shown that PNI is not a passive process occurring during tumor invasion but that neurogenic growth factors also induce nerve growth and innervation of the tumor (Demir et al., 2014; Bapat et al., 2016; Liu et al., 2022). The ablation of sensory neurons prevented PNI during carcinoma development and ultimately prolonged survival in a mice model (Saloman et al., 2016). It raises a concern about the roles of sensory neurons and Schwann cells, as they appear to transcend mere passive victims and instead actively constitute a significant stromal cell population that fosters cancer development (Demir et al., 2014; Deborde et al., 2016; Saloman et al., 2016). This naturally evokes an association between the sensory neurons and chronic pain in patients with bone cancer. PNI has been reported in several types of metastatic bone tumors such as prostate cancer, breast cancer, and hepatocellular carcinoma (Ciftci et al., 2015; Wang et al., 2015; Delahunt et al., 2020). However, the relationship between PNI and CIBP requires further studies to elucidate the underlying mechanisms. In summary, understanding the peripheral mechanism from the perspective of “vicious crosstalk” between sensory neurons and tumors in the bone microenvironment could help uncover more targets for pain management in CIBP.

### 3.2. Central mechanism

Chronic pain conditions, including CIBP, can lead to sensitization of the central nervous system (Lu et al., 2021). Central sensitization refers to the amplification of pain signaling within the spinal cord and brain, leading to increased pain perception. It is involved in the changes in reactive glial cell, synaptic transmission, neuronal excitability, and neuroplasticity (Latremoliere and Woolf, 2009). During this process, the nervous system becomes hypersensitive to pain signals, leading to increased pain sensitivity and development of chronic pain. Under CIBP conditions, central sensitization can occur via several mechanisms (Zajączkowska et al., 2019). One such mechanism involves the activation of glial cells in the spinal cord and brain. Glial cells, including microglia and astrocytes, are immune cells of the central nervous system that play key roles in neuroinflammation and pain processing (Inoue and Tsuda, 2018; Lu and Gao, 2023). In response to tumor growth and bone destruction, reactive glia releases proinflammatory cytokines and chemokines, which can sensitize nociceptors and contribute to central sensitization (Zhou et al., 2016). In addition to the release of proinflammatory cytokines and other signaling molecules, the microglia and astrocytes can interact to amplify the inflammatory response and promote central sensitization. For example, microglia release chemokines that recruit astrocytes to the site of inflammation, whereas astrocytes release cytokines that activate microglia and promote their survival and proliferation (Ji et al., 2019). Recent studies have shown that microglia and astrocytes undergo epigenetic changes in response to tumor growth and inflammation, which can lead to long-term changes in the gene expression and contribute to the development of chronic pain in CIBP (Wang et al., 2012; Vandenbark et al., 2021). For example, microglia and astrocytes can undergo DNA methylation and histone modification, which can alter the expression of genes involved in inflammation and pain signaling (Matias et al., 2018). The pain sensory system normally functions under a balance between excitation and inhibition. Down-regulation of K + -Cl--cotransporter-2 (KCC2) expression leads to these dysfunctions in spinal cord. A study reported by Zhao et al. (2023) demonstrated the activation of microglia through the BDNF–TrkB pathway affected neuronal KCC2 downregulation, contributing to dynamic allodynia induction in an SNI mouse model. Therefore, it is worth investigating those mechanism in CIBP condition. Another mechanism of central sensitization in CIBP involves the activation of N-methyl-D-aspartate (NMDA) receptors (Bennett, 2000). NMDA are ionotropic glutamate receptors involved in synaptic plasticity and pain processing. In response to persistent nociceptive inputs, NMDA receptors can become overactive, leading to central sensitization (Latremoliere and Woolf, 2009). In addition, central sensitization in CIBP can occur through the modulation of the descending pain pathways. Descending pain pathways originate in the brain and modulate pain signaling in the spinal cord. In response to persistent nociceptive input, the descending pain pathways can become dysregulated, leading to the development of chronic pain (Ossipov et al., 2014). Central sensitization is a complex mechanism involved in CIBP. Targeting central sensitization is a potential strategy for developing new therapies for this type of pain (Figure 1). However, further research is warranted to completely understand the mechanisms underlying central sensitization in CIBP and develop effective targeted therapies.

## 4. Clinical application

To manage moderate to severe pain, opioids are often recommended as first-line therapy in pain-relieving prescriptions, even though they are notorious for side effects, including the risk of addiction, respiratory depression, cognitive impairment, gastrointestinal reactions, and tolerance accompanied by opioid abuse (Baldo, 2021). The use of NSAIDs, gabapentinoids, antidepressants, paracetamol, or anticonvulsants may provide temporary and incomplete pain relief; however, their efficacy is frequently impeded by significant adverse effects (Scarborough and Smith, 2018).

Therefore, manipulating the activity of TRP channels and other potential ion channels using antagonists or agonists for inducing inactivation, blocking, and desensitization could be considered a new strategic approach for achieving analgesia in cancer pain. Capsaicin, a typical agonist, has been shown to activate TRP channels in various cancer cell lines, such as hepatoblastoma, small-cell lung cancer, and breast cancer, leading to apoptosis and reduced proliferation (Lau et al., 2014; Weber et al., 2016; Scheau et al., 2019). Capsaicin is widely used in the treatment of neuropathic, osteoarthritic, and musculoskeletal pain, and suppresses osteoclast formation, inflammatory bone resorption, and cyclooxygenase-2 (COX-2) expression; however, no clinical trials examining the potential role of capsaicin and its synthetic isomers or precursors in CIBP exist (Attal et al., 2006; Kobayashi et al., 2012; Boyd et al., 2019; Kolasinski et al., 2020). Only one phase 2 clinical trial (NCT03317613) has assessed the efficacy of the capsaicin patch in patients of cancer with neuropathic pain; however, it did not result in a concrete conclusion. Poor water solubility, irritation of the digestive system, and dangerous elevations in the body temperature limit the application of capsaicin as an oral analgesic (Gavva et al., 2008; Smutzer et al., 2018). Therefore, there is a need for improved delivery systems, carriers, and potential capsaicin analogs to overcome these side effects and inadequate efficacy.

Another capsaicin analog, resiniferatoxin (RTX), derived from *Euphorbia resinifera*, is the most potent known TRPV1 receptor agonist, surpassing both endogenous and synthetic compounds, and has been investigated as a potential therapeutic agent for cancer-induced pain (Bölcskei et al., 2010). Brown et al. (2005) reported the antinociceptive effects of intrathecal RTX in a canine bone cancer model. A similar result was obtained in a subsequent study that found that additional intrathecal administration of RTX provided effective pain relief and improved function in dogs with bone cancer without significant long-term side effects compared with standard analgesic therapy alone (Brown et al., 2015). An ongoing phase 1 study (NCT00804154) intrathecally evaluated the analgesic effect and safety on 45 participants. The participants are diagnosed with cancer, whose NRS score is greater than or equal to 6 and alternative methods of pain control are not sufficiently effective. Another completed phase 1b clinical trial (NCT03226574), carried out by Sorrento Therapeutics, established an escalation safety study to define the maximally tolerated dose of epidural RTX injection for the treatment of intractable pain associated with cancer. The company revealed on its website that 11 of 17 participants achieved a 30% decrease in pain based on NPRS scores (Moreau, 2021). Although the side effects were described as abnormal sensations that usually resolved over several hours, the clinical use of RTX needs to be proven in a large-scale study with a larger sample.

Several TRPV antagonists have been tested for analgesic effects. CPZ provides a multipronged approach for treating cancer pain in animal models, including CIBP from distal breast cancer metastases (Menéndez et al., 2006; Fazzari et al., 2015, 2017). However, a high effective dose, poor metabolic and pharmacokinetic properties, and nonspecific blockage of voltage-activated calcium channels other than TRPV1 have hindered subsequent clinical trials (Docherty et al., 1997; Kwak et al., 1998; Walker et al., 2003). SB-705498 is a selective TRPV1 antagonist that has been widely tested in clinical trials for acute migraine, chronic cough and toothache (clinicaltrials.gov). SB-705498 is safe and well tolerated for oral administration with no observed incidence of hyperthermia (Rami et al., 2006; Chizh et al., 2007). However, it does not show any advantage over the use of a placebo (Khalid et al., 2014). ABT-102 has been shown to have an acute antinociceptive effect in animal CIBP models. It exhibits good oral bioavailability and enhanced analgesic activity after repeated administration (Gomtsyan et al., 2008; Honore et al., 2009). In clinical trials, ABT-102 also demonstrated excellent bioavailability as a melt extrusion formulation, increasing the heat pain thresholds without intolerable hyperthermic events in participants (Rowbotham et al., 2011; Othman et al., 2012). Many other inhibited TRPV1 compounds, such as JNJ-17203212, JNJ-39439335, and JNJ-38893777, have been found to be effective in various pain models, and some, such as SB366791, demonstrate potential analgesic effects in bone cancer pain (Niiyama et al., 2009). Although these TRPV1 inhibitors have shown promise, they face a common challenge–the need for testing in large scale clinical trials. This is a crucial step toward establishing their safety and efficacy in humans. The next generation of TRPV1 antagonists with greater target selectivity and better penetration into the central nervous system is being designed and tested. One such compound, MDR-652, has shown promise as a potential TRPV1 antagonist, with improved selectivity and penetration into the central nervous system (Qiao et al., 2022).

## 5. Conclusion and future perspectives

In conclusion, ion channels play a critical role in CIBP and are involved in the transmission and modulation of pain signals, and their dysregulation can contribute to the development of chronic pain. Understanding the mechanisms underlying ion channel dysregulation in CIBP is important for the development of new therapies for CIBP. Future perspectives in this area of research include the development of novel ion channel modulators that selectively target specific ion channels involved in CIBP. Additionally, combination therapies targeting multiple ion channels and other mechanisms involved in CIBP may be more effective than those targeting a single ion channel.

## Author contributions

H-JL, X-BW, and Q-QW designed and wrote this review manuscript. All authors contributed to the article and approved the submitted version.

## Funding

This project was supported by National Natural Science Foundation of China (NSFC 32100806) and Natural Science Foundation of the Higher Education Institutions of Jiangsu Province (Grant No. 21KJB310010).

## Conflict of interest

The authors declare that the research was conducted in the absence of any commercial or financial relationships that could be construed as a potential conflict of interest.

## Publisher’s note

All claims expressed in this article are solely those of the authors and do not necessarily represent those of their affiliated organizations, or those of the publisher, the editors and the reviewers. Any product that may be evaluated in this article, or claim that may be made by its manufacturer, is not guaranteed or endorsed by the publisher.

## Abbreviations

CIBP: Cancer induced bone pain
DRG: Dorsal Root Ganglion
TRPV1: Transient Receptor Potential Vanilloid1
NaV1.7: voltage-gated sodium channel 1.7
TRPA1: transient receptor potential ankyrin 1
TRPM8: Transient receptor potential melastatin 8
TRPV4: Transient Receptor Potential Vanilloid 4
TRPV3: Transient Receptor Potential Vanilloid 3
CFA: Complete Freund’s adjuvant
ASICs: Acid-sensing ion channels
IL-1β: Interleukin-1β
IL-6: Interleukin 6
PGE2: Prostaglandin E2
CNS: Central Nervous System
NMDA: N-methyl-D-aspartate
NSAIDs: Non-steroidal anti-inflammatory drugs
COX-2: Cyclooxygenase-2
RTX: Resiniferatoxin
